# Immunologic mechanisms of seasonal influenza vaccination administered by microneedle patch from a randomized phase I trial

**DOI:** 10.1038/s41541-021-00353-0

**Published:** 2021-07-14

**Authors:** Nadine G. Rouphael, Lilin Lai, Sonia Tandon, Michele Paine McCullough, Yunchuan Kong, Sarah Kabbani, Muktha S. Natrajan, Yongxian Xu, Yerun Zhu, Dongli Wang, Jesse O’Shea, Amy Sherman, Tianwei Yu, Sebastien Henry, Devin McAllister, Daniel Stadlbauer, Surender Khurana, Hana Golding, Florian Krammer, Mark J. Mulligan, Mark R. Prausnitz

**Affiliations:** 1 Hope Clinic of the Emory Vaccine Center, Division of Infectious Diseases, Department of Medicine, School of Medicine, Emory University, Atlanta, Georgia; 2 Emory Vaccine Center, Department of Pediatrics, School of Medicine, Emory University, Atlanta, Georgia; 3 Laney Graduate School, Emory University, Atlanta, Georgia; 4 Micron Biomedical, Inc., Atlanta, Georgia; 5 Department of Microbiology, Icahn School of Medicine at Mount Sinai, New York, NY USA; 6 Division of Viral Products Center for Biologics Evaluation and Research, FDA, Silver Spring, MD USA; 7 New York University Langone Medical Center, Alexandria Center for Life Sciences, New York, NY USA; 8 School of Chemical & Biomolecular Engineering, Georgia Institute of Technology, Atlanta, Georgia

**Keywords:** Inactivated vaccines, Humoral immunity, Cellular immunity

## Abstract

In a phase 1 randomized, single-center clinical trial, inactivated influenza virus vaccine delivered through dissolvable microneedle patches (MNPs) was found to be safe and immunogenic. Here, we compare the humoral and cellular immunologic responses in a subset of participants receiving influenza vaccination by MNP to the intramuscular (IM) route of administration. We collected serum, plasma, and peripheral blood mononuclear cells in 22 participants up to 180 days post-vaccination. Hemagglutination inhibition (HAI) titers and antibody avidity were similar after MNP and IM vaccination, even though MNP vaccination used a lower antigen dose. MNPs generated higher neuraminidase inhibition (NAI) titers for all three influenza virus vaccine strains tested and triggered a larger percentage of circulating T follicular helper cells (CD4 + CXCR5 + CXCR3 + ICOS + PD-1+) compared to the IM route. Our study indicates that inactivated influenza virus vaccination by MNP produces humoral and cellular immune response that are similar or greater than IM vaccination.

## Introduction

Each year, seasonal influenza epidemics cause 3–5 million severe cases and 290,000 to 650,000 deaths globally^1^. More than half a million hospitalizations and 12,000 to 61,000 deaths occur in the United States alone^2^. Seasonal influenza vaccination remains the most effective way of preventing influenza infection. A better understanding of the immunologic mechanisms of influenza virus vaccines^3^ is needed to overcome their limited effectiveness (10–60%)^4^. Improving immune responses could be achieved by higher antigen doses^5^, use of adjuvants^6^, or alternative routes of immunization^7^.

Targeting the skin as a route of immunization may be better at stimulating B and T cells than traditional vaccination routes by utilizing the high density of dendritic cells and extensive microvascular and lymphatic networks facilitating migration of antigen-presenting cells to the regional lymph nodes^8^. Delivery to the skin has been effective for many vaccines; most notably, the use of scarification for smallpox vaccine resulted in the worldwide eradication of this highly virulent disease. In addition to the Bacille Calmette-Guérin vaccination against tuberculosis^9^, the intradermal route is also approved for the delivery of seasonal influenza virus vaccine, which allowed for antigen sparing^10,11^ and improvement of immunogenicity when equivalent doses of antigens have been compared (15 µg of HA per strain) between the intradermal and intramuscular (IM) routes^12^. Novel skin delivery modalities have targeted the vaccine delivery both intradermally and transdermally^13^. However, to date, the use of skin delivery systems has been hampered by the lack of appropriate vaccine delivery systems combining reliability, safety, and simplicity of use.

Microneedle patches (MNPs) are micron-scale, solid, conical structures made of dissolvable excipients or solid projections coated with vaccine that deliver vaccine antigens across the stratum corneum into the viable epidermis and dermis^14–18^. MNPs provide an alternative to traditional IM injection of influenza virus vaccine, especially for needle-phobic patients^19^ and offer multiple potential advantages^20,21^: simplicity (amenable to self-vaccination, distribution, and storage outside the cold chain, such as on the pharmacy shelf), potential for dose sparing, cost effectiveness (reducing costs of vaccine administration, cold chain, and sharps waste disposal)^22^, and safety (eliminating needle-stick injuries with non-sharps waste). When tested in animal models^23^ and compared to other systemic influenza virus vaccine delivery modalities, the MNPs can induce higher neutralizing antibody responses, higher protective immunity against lethal challenges of influenza viruses^24^, broader cross-reactive protection^25^, longer-lasting protection^25^, and dose sparing effect^26^.

In 2015–2016, a first-in-human, phase 1 clinical trial on the use of a dissolvable MNP for trivalent seasonal influenza virus immunization showed that the use of the MNPs was well tolerated, safe, had higher acceptability, and was strongly preferred by participants over conventional IM influenza vaccine administration^27^. The use of MNPs resulted in robust antibody responses measured by hemagglutination inhibition (HAI) that were at least as strong as IM injection for all vaccine strains, particularly for the influenza B virus strain. In that study, the immune response was characterized only in terms of HAI titers. Here, we report the exploratory results of a broader immunological analysis of the phase 1 clinical trial in a subset of participants comparing the humoral and cellular immunologic mechanisms of inactivated influenza virus vaccine administered either using MNPs or hypodermic needles.

## Results

### Study participants, demographics

From June 23rd to September 25th, 2015, 100 participants were enrolled and randomly assigned to study intervention. Detailed immunologic analyses were performed on the group receiving 2014–2015 inactivated influenza virus vaccine (IIV) by MNP (*n* = 11) versus IM route (*n* = 11), both delivered by a healthcare worker (Supplementary Fig. 1). The demographics of the groups were comparable: 50% of participants were female, 41% were non-white (Table 1). Ages ranged between 21 and 46, with a median of 26.5 years (IQR: 24.3, 32.0).

**Table 1:** Demographic characteristics of participants (*n* = 22)^a^.

Characteristic	MNP (*n* = 11)	IM (*n* = 11)
Age, years^b^	27.5 (4.76)	28.5 (5.16)
Sex, no. (%)		
Female	6 (55%)	5 (45%)
Male	5 (45%)	6 (55%)
Race, no. (%)		
White	6 (55%)	7 (64%)
Black	4 (36%)	3 (27%)
Other	1 (9%)	1 (9%)
Ethnicity, no. (%)		
Non-Hispanic	11 (100%)	9 (82%)
Hispanic	0 (0%)	2 (18%)
BMI, kg/m^2^	25.1 (4.11)	25.6 (5.15)
Prior IIV, no (%)		
2013–2014 season	4 (36%)	1 (9%)
2012–2013 season	1 (9%)	3 (27%)

^a^No statistical differences, assessed using Student’s *t* test for continuous variables and chi-square tests for categorical variables, were observed between the groups for any variable.

^b^Continuous variables are presented as mean (SD).

*BMI* body mass index, *IIV* inactivated influenza vaccine.

### Serological immune responses

**Fig. 1:**
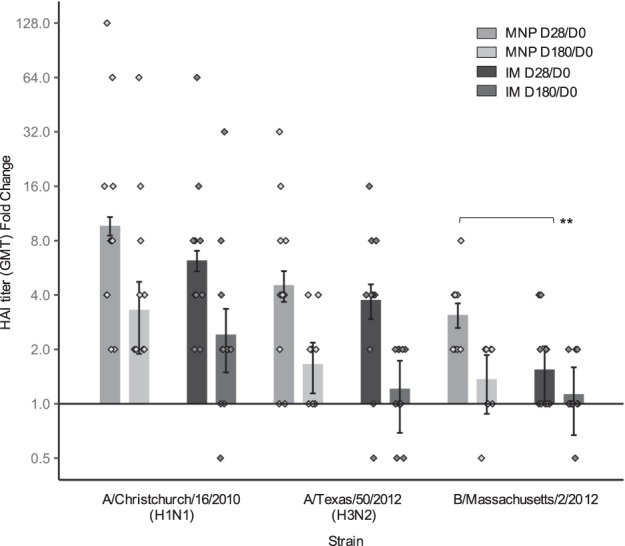
Fold change of hemagglutination inhibition (HAI) geometric mean titers (GMT) (log2) over baseline level at Day 28 and Day 180 following vaccination against A/Christchurch/16/2010, NIB-74 (H1N1), A/Texas/50/2012, NYMC X-223 (H3N2), B/Massachusetts/2/2012, NYMC BX-51(B) strains for MNP (*n* = 11) and IM (*n* = 11) groups with geometric standard error (vertical bars). Black lines indicate statistical significance based on Wilcoxon rank-sum test. **P < 0.01. IM, IIV by intramuscular route; MNP, IIV by microneedle patch; D, Day.

We found that HAI responses to MNP and IM immunization were similar at 1 and 6 months after vaccination, and that HAI responses were higher at 1 month than at 6 months (Fig. 1, Supplementary Table 1). There was no difference between the MNP and IM groups in HAI geometric mean titers (GMTs) for the H1N1, H3N2, and B strains at baseline or 1 and 6 months after vaccination. The geometric mean fold rise (GMFR) was higher for the B strain for the MNP group but not for other strains (*P* = 0.009). There was no difference in the Day 180-to-baseline GMT ratio between any strain or any group (H1N1: *P* = 0.33; H3N2: *P* = 0.29; B: *P* = 0.29). Seroprotection rates were similar between the groups with 100% at Day 28 for all strains and 82–100% when measured 6 months after vaccination (*P* = 1.00). Seroconversion at Day 28 for all strains varied between 55 and 82% for the MNP group and between 18 and 82% for the IM group, but these rates were not significantly different from each other (H1N1: *P* = 1.00; H3N2: *P* = 1.00; B: *P* = 0.18). The seroconversion dropped remarkably at Day 180 compared to Day 28 and was only present in some participants in the MNP group for H1N1 (46%) and H3N2 (18%) strains, and only in a few participants in the IM group only for the H1N1 strain (27%), but the differences between MNP and IM groups were not significant (*P* = 0.66). HAI responses in the substudy align with those previously observed in the complete study dataset^27^.

**Fig. 2:**
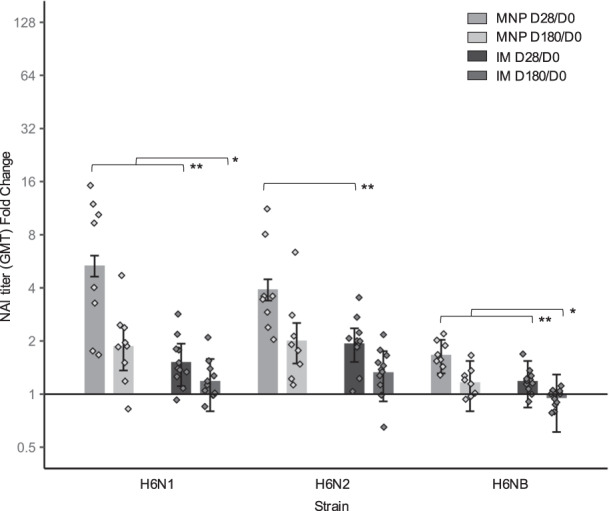
Fold change of neuraminidase inhibition (NAI) geometric mean titers (GMT) (log2) over baseline level at Day 28 and Day 180 following vaccination. Testing was performed against H6N1, H6N2, and H6NB strains for MNP (*n* = 8) and IM (*n* = 11) groups with geometric standard error (vertical bars). Black lines indicate statistical significance based on Wilcoxon rank-sum test. *P < 0.05; **P < 0.01. IM, IIV by intramuscular route; MNP, IIV by microneedle patch; D, Day.

While HAI has been routinely used to assess vaccine immunogenicity, lately neuraminidase inhibition (NAI) assays have been proposed as an independent correlate of protection^28,29^. Unlike HAI measurements, NAI responses were often different after MNP vaccination compared to IM (Fig. 2, Supplementary Table 2). At baseline, there was no difference between the groups in NAI GMTs, with the exception of the N1 strain where the GMT for the IM group was higher than the GMT for the MNP group (*P* = 0.04) (Supplementary Table 2). Interestingly, the fold changes between Day 28 and baseline for NAI GMTs were higher for N1 (*P* = 0.002), N2 (*P* = 0.003), and B (*P* < 0.001) strains after vaccination by MNP when compared to IM (Fig. 2, Supplementary Table 2)_._ These results were also observed at Day 180 (N1: *P* = 0.05; B: *P* = 0.02) with the exception of the N2 strain (*P* = 0.11).

**Fig. 3:**
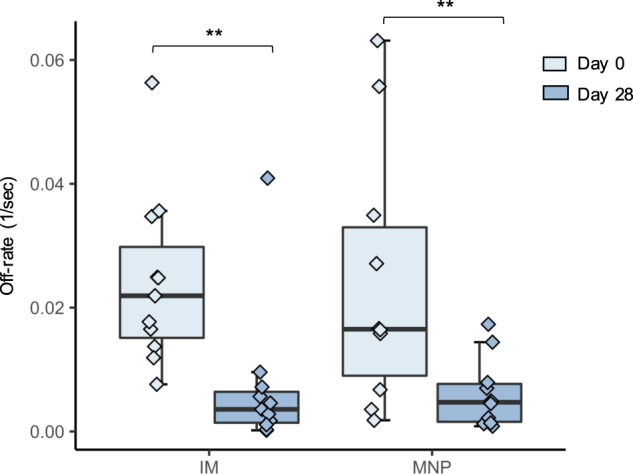
Changes in antibody binding affinity at Day 28 post vaccination by vaccine delivery group (MNP: *n* = 10; IM: *n* = 11). SPR analysis of human plasma from various vaccine cohorts was performed with functional rHA1. Plasma antibody off-rate constants were determined as described previously in “Methods”. The box portion of the plot represents the interquartile range (IQR: 25th percentile, median, and 75th percentile) of the data. Error bars represent smallest and largest values within 1.5 times IQR. Black lines indicate statistical significance based on Wilcoxon rank-sum test. *P < 0.05; **P < 0.01. IM, IIV by intramuscular route; MNP, IIV by microneedle patch.

We also measured antibody affinity by analyzing the antigen–antibody complex dissociation kinetics (off-rates) bound to H1N1pdm09 HA1 (globular head). Antibody affinity could be of clinical significance as high levels of low avidity antibodies in infected individuals were associated with severe H1N1pdm09 disease^30^. Pre-vaccination off-rates varied among participants ranging between 10^−2^ s^−1^ and 10^−3^ s^−1^ (Fig. 3) and were comparable between groups (*P* = 0.65). Increase in binding affinities was observed within both the MNP and IM groups at Day 28 post vaccination when compared to baseline (*P* = 0.002; *P* = 0.01, respectively).

### Cytokines/chemokines

**Fig. 4:**
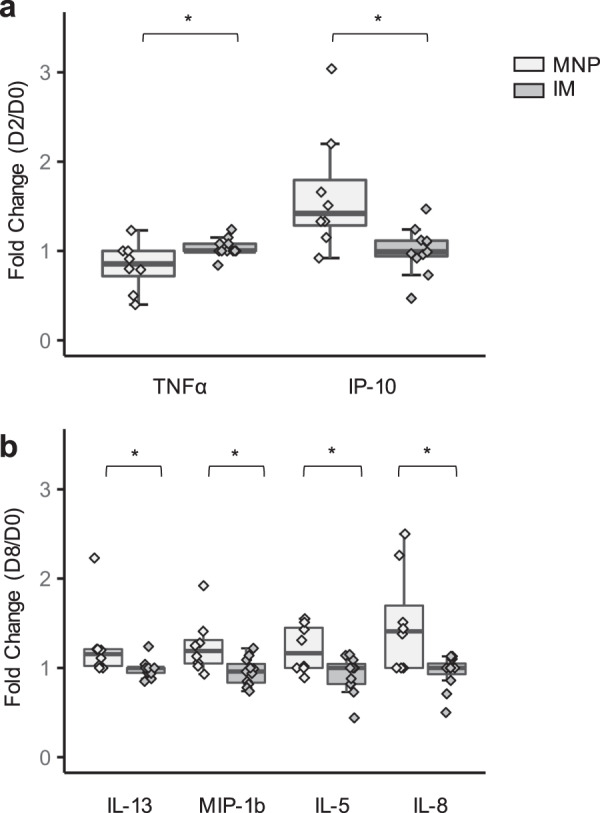
Statistically significant fold changes in cytokines/chemokines following vaccination. Fold changes at **a** Day 2 and **b** Day 8 post vaccination by vaccine delivery group (MNP: *n* = 8; IM: *n* = 11). The proteins were detected by antibody-bound beads and quantitated using a Luminex instrument. The box portion of the plot represents the interquartile range (IQR: 25th percentile, median, and 75th percentile) of the data. Error bars represent smallest and largest values within 1.5 times IQR. Black lines indicate statistical significance based on Wilcoxon rank-sum test. *P < 0.05; **P < 0.01. IM, IIV by intramuscular route; MNP, IIV by microneedle patch.

An increase was seen in IP-10 (*P* = 0.01) for the MNP group compared to the IM group when measured on Days 2–3 after immunization and compared to baseline. However, for TNF-α (*P* = 0.04), the level was higher for the IM group compared to the MNP group at Days 2–3 when compared to baseline. When Day 8 values were compared to baseline for the following cytokines: IL-8 (*P* = 0.02); IL-5 (*P* = 0.034); IL-13 (*P* = 0.013) and MIP-1b (*P* = 0.016) they were all higher for the MNP group (Fig. 4). After adjusting for multiple comparisons via Bonferroni correction, none of these findings remain statistically significant. Other changes in cytokines and chemokines over baseline values did not differ significantly between groups, including IL-6 and MIP-1 alpha.

### Cellular immune responses

**Fig. 5:**
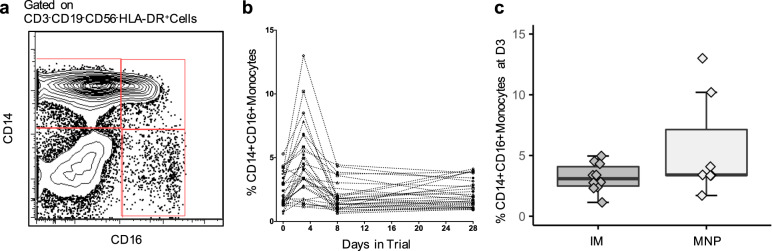
CD14 + CD16+ monocytes after vaccination. **a** Monocytes were identified within the SSC-A-hi FSC-A-hi cells as the CD3-CD19-CD20-CD56- HLA-DR+ population and gated for the CD14 + CD16-, CD14 + CD16+, and CD14dimCD16++ subsets. **b** Temporal CD14 + CD16+ monocytes frequency over CD3-CD19-CD56-HLA-DR+ cells at baseline, 2–3, 8, and 28 days post vaccination. **c** Percentage of CD14 + CD16+ monocytes at Days 2–3 on CD3-CD19-CD56-HLA-DR+ cells by vaccine delivery group (MNP: *n* = 7; IM: *n* = 10). The box portion of the plot represents the interquartile range (IQR: 25th percentile, median, and 75th percentile) of the data. Error bars represent smallest and largest values within 1.5 times IQR. IM, IIV by intramuscular route; MNP, IIV by microneedle patch.

Proinflammatory monocytes in blood were defined as CD14 + CD16+ monocytes at Days 2–3 within the CD3-CD19-CD56-HLA-DR+ cell subset. No significant difference was observed between the groups (*P* = 0.33) (Fig. 5).

**Fig. 6:**
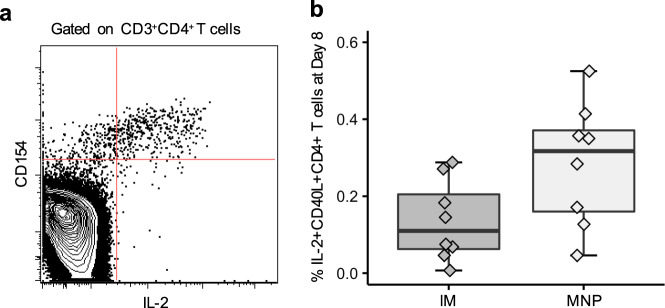
IL-2 secreting CD4+ T cells after vaccination. **a** Representative FACS plot showing CD3 + CD4+ T cells expressing CD154 (CD40L) and IL-2 by ICS assay. **b** Percentage of vaccine-induced IL-2 + CD40L + CD4+ T cells at Day 8 by vaccine delivery group (MNP: *n* = 8; IM: *n* = 8). The box portion of the plot represents the interquartile range (IQR: 25th percentile, median, and 75th percentile) of the data. Error bars represent smallest and largest values within 1.5 times IQR. IM, IIV by intramuscular route; MNP, IIV by microneedle patch.

We looked at polyfunctional CD4+ T cells producing different cytokines such as IL-2, IL-21, IFN-γ, and CD154 (CD40L). Relative to baseline, there was a numerical increase in the median percentage of vaccine-induced IL-2 + CD40L + CD4+ T cells at Day 8 in the MNP group [0.32% (IQR: 0.16, 0.37)] compared to the IM group [0.11% (IQR: 0.06, 0.21)], but it was not statistically significant (*P* = 0.07) (Fig. 6). No significant change of IFN-gamma, TNF-alpha, and IL-21 was detected over baseline.

**Fig. 7:**
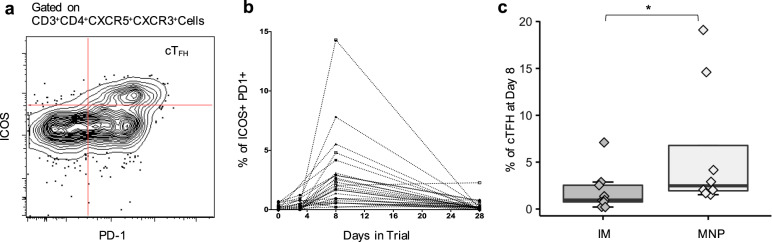
Circulating T follicular helper (cT_FH_) cells after vaccination. **a** Representative FACS plot showing ICOS and PD-1 co-expressing CD3 + CD4 + CXCR5 + CXCR3 + cT_FH_ cells. **b** Temporal cells at baseline and days 2–3, 8, and 28 post vaccination. **c** Percentage of cT_FH_ (CD4 + CXCR5 + CXCR3 + ICOS + PD-1+) at Day 8 post vaccination by vaccine delivery group (MNP: *n* = 8; IM: *n* = 9). The box portion of the plot represents the interquartile range (IQR: 25th percentile, median, and 75th percentile) of the data. Error bars represent smallest and largest values within 1.5 times IQR. Black lines indicate statistical significance based on Wilcoxon rank-sum test. *P < 0.05; **P < 0.01. IM, IIV by intramuscular route; MNP, IIV by microneedle patch.

Prior studies have shown a transient increase in cT_FH_ cells measured 7 days after influenza virus vaccination correlating with the emergence of antibody-secreting cells, increased levels of serum antibody titers and affinity, as well as memory B cell responses^31–33^. These T cells express the chemokine receptor CXCR5 and co-stimulatory molecules ICOS and PD-1, and thus belong to a circulating compartment of T_FH_ cells. There was a higher percentage of cT_FH_ (CD4 + CXCR5 + CXCR3 + ICOS + PD-1+) at Day 8 post vaccination in the MNP group [2.48% (IQR: 1.9, 6.8))] compared to the IM group [0.96% (IQR: 0.8, 2.5)] (*P* = 0.04) (Fig. 7).

**Fig. 8:**
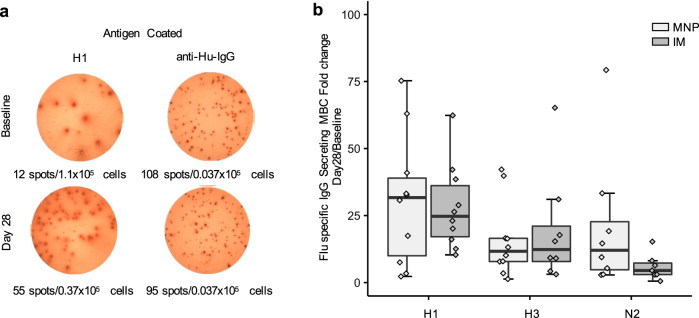
Influenza-specific IgG-secreting memory B cells (MBCs) after vaccination. **a** Representative ELISpot showing H1 protein and IgG protein-specific memory B cells at baseline and Day 28 post vaccination. **b** Fold change of influenza-specific IgG-secreting Memory B Cells (MBCs) responses at Day 0 and Day 28 by vaccine delivery group (MNP: *n* = 10; IM: *n* = 11). The box portion of the plot represents the interquartile range (IQR: 25th percentile, median, and 75th percentile) of the data. Error bars represent smallest and largest values within 1.5 times IQR. IM, IIV by intramuscular route; MNP, IIV by microneedle patch.

Influenza virus-specific immunoglobulin G (IgG)-secreting memory B cells (MBCs) responses were measured at baseline and at Day 28 without any significant difference between the groups for all 3 antigens: H1 (*P* = 0.91), H3 (*P* = 0.55), and N2 (*P* = 0.15) (Fig. 8).

## Discussion

MNPs have the potential to offer many benefits to immunization programs including decreased transport and distribution costs (thermostable, small footprint), ease and safety of administration (no need for reconstitution, no specialized skills), and greater acceptability and potentially less hesitancy by end-users^17^. While immunologic advantages of MNPs as a novel vaccine delivery method have been demonstrated in animal models, the current study is one of few to highlight the unique immunologic features of MNPs in humans in the setting of influenza virus vaccination, other than dose sparing^18,34^.

Immune responses induced by available IIV vaccines primarily target the immunodominant globular head domain of the HA which is responsible for viral attachment and fusion. The HAI assay, measuring the antibodies to the HA head, is considered a surrogate correlate of protection and used in the evaluation of vaccines for licensure^35^; however, an HAI titer of 40 only provides a 50% putative protective threshold in adults^36^. In addition, HA head-specific antibodies do not reflect the whole spectrum of protective antibodies. Blocking neuraminidase (NA), another major surface glycoprotein, is an effective way to inhibit viral infection replication and shedding^37^. Antibodies against NA have been shown to confer protection from natural influenza virus infection^28,29^ as well as in an influenza challenge model^38^ and to shorten the duration of shedding and symptoms^39^. Therefore, NAI assay is being considered as an independent correlate of protection^40,41^. Interestingly, the MNPs were able to induce a better NAI responses at Days 28 and 180 when compared to baseline in comparison to the IM route.

Intradermal vaccination directly targets epidermal Langerhans cells and dermal dendritic cells, which are essential for efficient cellular and humoral responses^42^. In our study, MNPs showed an early increase in IP-10^43^, MIP-1b, and IL-8 compared to baseline that was statistically higher than the IM route with a trend toward a higher proinflammatory monocyte responses 2–3 days after vaccination. Interestingly, higher levels of allergic inflammation IL-5 and IL-13 were noted in the MNP group, which could explain the local transient pruritus reported by a subset of vaccinees^27^.

Though antibodies have been the hallmark of protection against influenza vaccines and infection, CD4+ T cells confer protection too^44,45^. Pre-existing CD4+ T cells result in lower virus shedding and less severe influenza symptoms in experimental A/H3N2 human challenge^44^ and natural infection with A/H1N1pdm^45^ in the absence of protective antibodies. In our study, MNPs did not lead to a better HA-specific IL-2 + CD154(CD40L) + CD4+ T cells response at Day 8 relative to baseline when compared with the response seen with IIV administered IM.

In addition, the interactions between CD4+ T cells and B cells promote both extrafollicular responses that generate short-lived, class-switched antibody-secreting cells as well as germinal center reactions through cT_FH_ that result in long-lived, affinity-matured antibody-secreting cells and memory B cells^46^.

There are limitations to this study. A major limitation is the small group sizes typical for a phase 1 study and availability of samples. The lack of difference in HAI GMT between the different delivery routes could have been due to pre-existing antibodies linked to reduced humoral and effector B cell responses after vaccination^47,48^ making it difficult to compare our results with other studies where the majority of the participants had lower pre-existing antibodies^18,34^. Detecting differences in immune profiles between the different routes of delivery might have been easier in naive individuals (young children)^49^, where strong immunologic advantages of influenza vaccination by MNP compared to the IM route have been seen in a young mouse model^50^.

There was a difference between the HA quantity in the vaccines delivered by IM versus MNPs, with 15–37% lower quantities delivered by MNPs^27^. Despite this, MNP delivery still showed similar HAI as well as superior NAI and cT_FH_ cell responses highlights another advantage of MNPs with regard to antigen sparing. While the HA quantity in seasonal influenza virus vaccine is at least 15 µg per strain, the quantity and quality of NA included in the vaccine are not standardized^51^. We measured the quantity of HA administered by the MNPs, but did not measure the quantity of NA in either vaccine type, though both the IM doses and the monobulk used to prepare the MNPs were provided by the manufacturer, so we would expect the ratio of HA-to-NA to be the same in MNP and IM formulations.

Memory B cell responses in our study were measured early, 28 days after vaccination, and did not show any difference between the groups; assessing these responses at a later timepoint (4–6 months) may have revealed differences in the groups. In addition, CD8+ T cells play an important role in influenza virus immunity. Due to limitation in availability of peripheral blood mononuclear cells (PBMCs) in our samples, we were unable to conduct these assays.

In addition, an extended immunological analysis was not performed on any subject who received the placebo MNP in the trial. It is not possible therefore to determine if any of the responses observed were due to the application of the dissolving MNP rather than being due to delivery of the influenza antigen.

In summary, the use of MNPs for influenza virus vaccination offers both logistical advantages as well as promising immunologic advantages reported here and elsewhere. Our study showed higher cT_FH_ cells along with higher NA function in participants vaccinated with MNPs when compared to the traditional IM needle and syringe.

## Methods

### Participants

The partially blinded, randomized, placebo-controlled, phase 1 study recruited 100 healthy non-pregnant, immunocompetent adults aged 18–49 years who had not previously received the influenza virus vaccine during the 2014–15 influenza season. Additional inclusion and exclusion criteria are detailed on ClinicalTrials.gov (NCT02438423; date of registration: May 8, 2015) and have been previously described elsewhere^27^.

### Randomization and study groups

Participants, who consented for the substudy, were randomly assigned to receive either IIV by MNP or by IM injection, in each case administered by a healthcare worker. In addition, there was a group that received placebo by MNP and another group that received IIV by MNP self-administered by study participants. As the main objective of the phase 1 trial was to compare IIV vaccination by MNP and IM, both applied by healthcare worker, the substudy compared these two groups for detailed immunologic analysis (Supplementary Fig. 1). Laboratory testing was performed in a blinded manner.

### Vaccines

The licensed 2014–15 seasonal trivalent IIV (Fluvirin) was kindly provided by Seqirus (Cambridge, MA) in single-dose, prefilled syringes for IM injection containing the following three influenza vaccine strains: A/Christchurch/16/2010, NIB-74 (H1N1); A/Texas/50/2012, NYMC X-223 (H3N2); and B/Massachusetts/2/2012, NYMC BX-51(B). The MNPs were designed at the Georgia Institute of Technology (Atlanta, GA) and manufactured by the Global Center for Medical Innovation (Atlanta, GA) under Phase 1 Good Manufacturing Practice. The antigen incorporated into the MNPs was also provided by Seqirus and comprised the same three vaccine strains. The formulation and fabrication methods have been previously described^52^.

IM was administered by hypodermic needle in the deltoid muscle of the arm preferred by the participant and the MNPs were manually applied to the dorsal aspect of the wrist of the non-dominant arm and left on the skin for 20 min.

### Assessment of vaccine delivery

The difference between the antigen content measured by single radial immunodiffusion (SRID) assay of unused MNPs (i.e., 18 μg hemagglutinin (HA) per vaccine strain) and that of the residual antigen content of the 11 MNPs used in the substudy showed that the mean HA dose delivered by MNPs was 10.8 μg (Standard Error (SE) 1.1) for the H1N1 strain, 14.4 μg (SE 0.8) for the H3N2 strain, and 13.3 μg (SE 0.6) for the B strain^27^. Our measurements also showed that the IM injection administered 18 μg of HA per H1N1 vaccine strain, 17 μg of HA per H3N2 vaccine strain, and 15 μg of HA per B vaccine strain with a delivery efficiency of 60%, 85%, and 89%, respectively. Mean dose delivered by each strain significantly differed between MNP and IM groups (H1N1: *p* < 0.001; H3N2: *p* = 0.013; B: *p* = 0.018).

### Blood samples

Blood samples for the substudy were collected at Days 0 (pre-vaccination), 2–3, 8–10, 26–30, and 166–194 days post vaccination. Peripheral blood mononuclear cells (PBMCs) were separated from sodium citrate whole blood using a Ficoll-Hypaque gradient and stored in a liquid nitrogen tank. Cryopreserved PBMCs were thawed in a 37 °C water bath and washed. Before use, cells were counted and checked for viability by Trypan blue dye exclusion.

### Hemagglutination inhibition assay

We used reference strains contained in the 2014–15 trivalent influenza virus vaccine: H1N1 virus reference strain A/Christchurch/16/2010 (#10/216, National Institute for Biological Standards and Control, Potters Bar, Hertfordshire, UK), H3N2 A/Texas/50/2012 (#FR1210), and B virus reference strain B/Massachusetts/2/2012 (#FR1196, Influenza Reagent Resource of the Centers for Disease Control and Prevention, Atlanta, GA, https://www.internationalreagentresource.org). Influenza viruses were propagated in Madin-Darby Canine Kidney (MDCK).2 cells and MDCK.2 SIAT1 cells in the presence of tosylamide-2-phenylethyl chloromethyl ketone-treated trypsin (Sigma-Aldrich, St Louis, MO). HAI assays were previously performed and described^27^.

### Neuraminidase inhibition assay

H6NX reassortant influenza A viruses were generated by reverse genetics as described previously^53^. These viruses contain the HA (H6) gene from influenza A/turkey/Massachusetts/3740/1965 (H6N2) virus, gene segments encoding internal proteins from influenza A/Puerto Rico/8/1934 (H1N1) virus, and one of the following NA gene segments: N1 of A/California/04/2009 (H1N1); N2 of A/Texas/50/2012 (H3N2) or influenza B NA of B/Yamagata/16/1988^54^. H6NX viruses were propagated in 10-day-old specific-pathogen-free embryonated chicken eggs at 37 °C for 2 days.

To determine the ideal stock virus concentration to be used in the NAI assay, an enzyme-linked lectin assay (ELLA) was first performed for all reassortant H6NX viruses. In brief, 96-well microtiter plates (Thermo Fisher, Waltham, MA) were coated with 100 μL/well fetuin (Sigma) at a concentration of 25 μg/mL in PBS and stored at 4 °C. The next day, the plates were washed three times with T-PBS [PBS containing 0.1% Tween-20 (Sigma)] and blocked in 200 μL blocking buffer (5% bovine serum albumin (BSA) (MP Biomedicals, Irvine, CA) in PBS) at room temperature for at least 1 h. On a separate cell culture 96-well plate, virus was serially diluted 1:2 in PBS and added to the fetuin-coated plate. The plates were incubated for 2 h at 37 °C and washed four times with T-PBS. Peanut agglutinin conjugated to horseradish peroxidase (PNA-HRP, Sigma) at a concentration of 5 µg/mL diluted in PBS was added to the plate (100 µL per well) and incubated in the dark for 1.5 h at room temperature. The plate was developed with 100 µL o-phenylenediamine dihydrochloride (SigmaFast) and the reaction stopped after 5 min with 50 μL 3 M hydrochloric acid (Thermo Fisher). The plate was read at a wavelength of 490 nm with a microtiter plate reader (Bio-Tek, Winooski, VT). The data were fitted to a nonlinear regression curve (four parameters) to determine the effective concentration 50 (EC_50_) of the different viruses.

Working stocks of each H6NX virus were prepared by diluting the virus stock to two times the EC_50_.

To perform the NAI assays, the microtiter plates were coated and blocked as described above. In the first row of a cell culture 96-well plate, 150 µl of 1:20 pre-diluted and pre-treated human serum was added. The human sera were pre-treated with RDE (Denka Seiken, Tokyo, Japan) to reduce non-specific background inhibition. In all other wells, 75 µl PBS was added. The sera were serially diluted two-fold by transferring 75 µl into the next row. Then, 75 μL of virus working stock (2× EC_50_) was added to wells of the serially diluted human sera and incubated for 1.5 h on a shaker. The fetuin-coated plates were washed three times with T-PBS and the virus/serum mixture was added to the plates and incubated for 2 h at 37 °C. The remainder of the assay was performed as described above. The data were fitted to a nonlinear regression curve (four parameters) and the inhibitory concentration 50 (IC_50_) was calculated for all human serum samples individually. Sample sizes available for statistical analysis differed based on sample availability (MNP: *n* = 8; IM: *n* = 11).

### Serum antibodies binding assay

The DNA gene segments corresponding to the HA1 proteins of A/California/07/2009 (H1N1pdm09) virus were cloned as *Not*I-*Pac*I inserts into a T7 promoter based pSK expression vector in which the desired polypeptide can be expressed as a fusion protein with His_6_ tag at the C-terminus. *E. coli* Rosetta Gami cells (Novagen, Madison, WI) were used for expression of HA1 proteins. Following expression, inclusion bodies were isolated by cell lysis and multiple washing steps, denatured, refolding in redox-folding buffer, and dialyzed. The dialysate was filtered through a 0.45 µm filter and purified by HisTrap Fast flow chromatography (information on instrument/materials). The purified proteins were characterized by the presence of oligomers by gel-filtration chromatography and by functional binding to turkey RBCs in a hemagglutination assay.

To confirm that the rHA1 proteins formed oligomers (similar to the native spike HAs on virions) hemagglutination assay was performed with human RBCs. The HA0 from H1N1pdm09 and the purified rHA1 proteins agglutinated RBC to various concentrations. We previously demonstrated that several recombinant HA1 domains produced using bacterial system resembled native viral HA in EM, formed functional trimers/oligomers that were fully immunogenic, generated high-affinity antibodies, and protected ferrets from influenza challenge with pandemic strains^55–58^. The HA1 domains could also adsorb the majority of neutralizing antibodies from post-vaccination polyclonal antibodies in human plasma.

Steady-state equilibrium binding of post-vaccination sera was monitored at 25 °C using a ProteOn surface plasmon resonance biosensor (SPR, Bio-Rad, Hercules, CA). The rHA1 protein is coupled to a GLC sensor chip with amine coupling with 500 resonance units (RU) in the test flow cells. Samples prepared at 10-, 50-, and/or 250-fold dilutions were injected at a flow rate of 50 µL/min (120-s contact time) for association, and dissociation performed over a 600 s interval (at a flow rate of 50 µL/min). Responses from the protein surface were corrected for the response from a mock surface and for responses from a separate, buffer-only injection. MAb 2D7 (anti-CCR5) was used as a negative control. Antibody off-rate constants, which describe the fraction of antigen–antibody complexes that decay per second, were determined directly from the plasma sample interaction with rHA1 using SPR in the dissociation phase only for the sensorgrams with Max RU in the range of 20–150 RU and calculated using the BioRad ProteOn manager software for the heterogeneous sample model as described before. Off-rate constants were determined from two independent SPR runs. Sample sizes available for statistical analysis differed based on sample availability (MNP: *n* = 10; IM: *n* = 11).

### Innate and T-cell phenotyping assays

Monoclonal antibodies used for staining of PBMCs for innate cell phenotyping were CD3 (UCHT1, #557943), CD19 (HIB19, #557921), CD14 (M5E2, #565283), HLA-DR (G46-6, #560651), CD11c (O33-782, #561355), and CD123 (7G3,554529) from BD Biosciences (Franklin Lakes, NJ), CD56 (MEM188,17-0569) and CD16 (CB16,47-0168) from eBiosciences (San Diego, CA); for cT_FH_ phenotyping CD3 (SP34-2, #562877), CD4 (L200, #560836), CXCR5 (RF8B2), and CXCR3 (IC6/CXCR3) BD Biosciences, ICOS (C398.4 A) and PD-1 (EH12.2H7) from Biolegend (San Diego, CA) (Supplementary Fig. 2). Data were collected on an LSRII (BD Biosciences) and data were analyzed using R 3.6.3 software. Sample sizes available for statistical analysis differed based on sample availability (monocytes—MNP: *n* = 7; IM = 10, cT_FH_—MNP: *n* = 8; IM: *n* = 9).

### Influenza virus-specific CD4+ T cells by intracellular cytokine assay (ICS)

Peptide pools consisting of 15-mers with 11 amino acid overlaps spanning 3 HA proteins in the vaccine were synthesized (GenScript, Piscataway, NJ). Cryopreserved PBMCs were thawed in a 37 °C water bath and washed. Cells were counted and checked for viability by Trypan blue dye exclusion and rested overnight at incubator 37 °C with 5% CO_2_ and then incubated with virus peptide pools at final concentrations of 2 μg/mL of each peptide in the presence of CD28 and CD49d (BD Biosciences, #340957 and #340976). Negative control samples were left un-stimulated in culture medium, and positive control samples were treated with *Staphylococcus* enterotoxin B (Sigma) at a final concentration of 1 μg/mL. Cells were cultured for 2 h at 37 °C, and then a cocktail containing brefeldin A and monensin was added (eBioscience, #004980-93), followed by a 4 h culture. Cells were washed with 1xPBS; surface stained with Aqua live/dead stain L423102 (Biolegend), and then fixed and permeabilized using a Cytofix/Cytoperm kit (BD Biosciences; 554722). The cells were then stained with the following fluorescence conjugated antibodies: CD3(SP34-2), CD4(L200), IL-2 (MQ1-17H12, #554567), and TNF-α (MAB11, #550679) from BD Biosciences; and IFN-γ (4 S.B3, #47-731942) and IL-21 (2A3-N2) from eBioSciences. After the cells were washed, data were collected on an LSRII Fortessa instrument (BD Biosciences). Compensation was performed using tubes of CompBeads (BD Biosciences, # 51-90-9001229) individually stained with each fluorophore and compensation matrices were calculated with FACSdiva. Data were analyzed using Flowjo software. Sample sizes available for statistical analysis differed based on sample availability (MNP: *n* = 8; IM: *n* = 8).

### Memory B cell analysis by enzyme-linked immune absorbent spot (ELISpot) assay

Recombinant influenza virus HA proteins of A/Christchurch/16/2011 (H1N1) pdm09 (NR-42487) and neuraminidase (NA) Protein from A/Brisbane/10/2007 (H3N2) (NR-43784) were provided by BEI Resources (Manassas, VA). HA of A/Victoria/361/2011/H3N2 (FR-1059) was provided by the Centers for Disease Control and Prevention International Reagent Resource (Manassas, VA). Cryopreserved PBMCs were thawed and checked for viability as described above. Memory B cell assays were performed as previously described^59^. In brief, PBMCs were plated in 24-well dishes at 5 × 10^5^ cells/well in R-10 medium supplemented with IL-2 and R488 (CTL-hBPOLYS-200, CTL) plus antigens for 6 days. For ELISpot, 96-well filter plates (Millipore, #MSHAN4B50) were coated 0.1 µg per well of HA proteins or 1 µg per well of anti-human IgG. The plates were left to adsorb overnight at 4 °C, washed four times in PBS, and incubated in Roswell Park Memorial Institute (RPMI) medium with 10% (vol/vol) fetal bovine serum (FBS) for 1 h at 37 °C. RPMI was removed, and PBMCs suspended in RPMI with 10% FBS were placed in each well with threefold dilutions. Plates were incubated at 37 °C overnight, then were washed twice with PBS followed by four times in PBS with Tween® 20 (PBST) and incubated for 2 h at room temperature with biotinylated anti-human IgG (Jackson Immunoresearch Laboratory, #709065098) diluted 1:1000 in PBST with 2% FBS. Plates were washed four times in PBST and incubated for 1 h at room temperature with streptavidin-HRP (Vector Laboratories #A 2004) diluted 1:1000 in PBST with 2% FBS. Plates were washed four times each in PBST, then with PBS, followed with incubation with 3-Amino-9-ethylcarbazole substrate kit (EMD Millipore Corporation, Substrate #152226, Buffer #152224) for 10 min until spot development. Plates were washed with water and allowed to dry; images were obtained using a CTL ELISpot plate reader. Data are presented as the percentage of influenza virus-specific IgG-secreting cells among total IgG-secreting cells. Sample sizes available for statistical analysis differed based on sample availability (MNP: *n* = 10, IM: *n* = 11).

### Cytokines and chemokines

Cytokines tested included IL-1β, IL-1RA, IL-2, IL-2R, IL-4, IL-5, IL-6, IL-7, IL-8, IL-10, IL-12p40/p70, IL-13, IL-15, IL-17, TNF-α, IFN-α, IFN-γ, GM-CSF, MIP-1α, MIP-1β, IP-10, MIG, Eotaxin, RANTES, and MCP-1. Human 25-plex cytokine kits (Cat # LHC0009M) were purchased from Thermo Fisher (Waltham, MA) for Luminex assays and used according to the manufacturer’s recommendations with modifications. Beads were added to a 96-well plate and washed in a Biotek ELx405 washer. Samples were added to the plate containing the mixed antibody-linked beads and incubated at room temperature for 1 h followed by overnight incubation at 4 °C. Cold and room temperature incubation steps were performed on an orbital shaker at 500–600 rpm. Following the overnight incubation, plates were washed in a Biotek ELx405 washer and then biotinylated detection antibody added for 75 min at room temperature with shaking. Plates were washed again as above and streptavidin-PE was added. After incubation for 30 min at room temperature, wash was performed as above and reading buffer was added to the wells. Each sample was measured in duplicate. Plates were read using a Luminex LX 100 instrument with a lower bound of 50 beads per sample per cytokine. As cytokine changes are observed early after vaccination, we measured cytokine levels 2–3 days and 8–10 days after vaccination and compared them to pre-vaccination levels. Sample sizes available for statistical analysis differed based on sample availability (MNP: *n* = 8, IM: *n* = 11).

### Statistics

Descriptive statistics of the population at enrollment were compared using Student’s *t* test for continuous variables and chi-square tests for categorical variables. The immunogenicity population included all participants who provided serum samples at baseline and at least 28 or 180 days after study product administration (MNP: *n* = 11, IM: *n* = 11). GMT of HAI and NAI antibodies were determined for each strain and 95% confidence intervals (CI) were based on the normal distribution of log-transformed data. Mann–Whitney Wilcoxon tests were used to compare MNP and IM groups. We adjusted for multiple comparisons using Bonferroni correction.

The proportion of participants achieving seroprotection (defined as a HAI antibody titer of 1:40 or greater) and seroconversion (defined as a minimum four-fold increase in post-vaccination HAI and NAI antibody titer) ~28 days following receipt of study products were determined for each strain in the MNP and IM groups. Clopper-Pearson exact confidence intervals were calculated, and chi-square tests were used to compare frequencies of seroprotection and seroconversion between each group. For all cellular assays, median percentage and IQR were reported and Mann–Whitney Wilcoxon tests were used to compare MNP and IM groups.

All statistical analyses were performed using the R statistical software version 3.6.3 and a two-sided *p*-value of <0.05 was considered statistically significant.

### Study approval

All participants provided written informed consent for participation in the study before enrollment. The study was approved by the Emory University and the Georgia Institute of Technology institutional review boards.

## Supplementary information

Supplementary Information

## Data Availability

The data that support the findings from this study are available from the corresponding author on reasonable request.

## References

[1] World Health Organization. *Influenza (seasonal) - Ask the expert; Influenza Q&A*, http://www.who.int/news-room/fact-sheets/detail/influenza-(seasonal) (2020).

[2] Centers for Disease Control and Prevention. *Disease Burden of Influenza*, accessed June 4th, 2021 https://www.cdc.gov/flu/about/burden/index.html.

[3] Erbelding EJ (2018). A universal influenza vaccine: the strategic plan for the National Institute of Allergy and Infectious Diseases. J. Infect. Dis..

[4] Centers for Disease Control and Prevention. *CDC Seasonal Flu Vaccine Effectiveness Studies*, accessed February 6th, 2021 https://www.cdc.gov/flu/vaccines-work/effectiveness-studies.htm.

[5] Robertson CA (2016). Fluzone® high-dose influenza vaccine. Expert Rev. Vaccines.

[6] O’Hagan DT (2007). MF59 is a safe and potent vaccine adjuvant that enhances protection against influenza virus infection. Expert Rev. Vaccines.

[7] Robertson CA, Tsang P, Landolfi VA, Greenberg DP (2016). Fluzone® intradermal quadrivalent influenza vaccine. Expert Rev. Vaccines.

[8] Nicolas JF, Guy B (2008). Intradermal, epidermal and transcutaneous vaccination: from immunology to clinical practice. Expert Rev. Vaccines.

[9] Franco-Paredes C, Rouphael N, del Rio C, Santos-Preciado JI (2006). Vaccination strategies to prevent tuberculosis in the new millennium: from BCG to new vaccine candidates. Int. J. Infect. Dis..

[10] Belshe RB (2004). Serum antibody responses after intradermal vaccination against influenza. N. Engl. J. Med..

[11] Kenney RT, Frech SA, Muenz LR, Villar CP, Glenn GM (2004). Dose sparing with intradermal injection of influenza vaccine. N. Engl. J. Med..

[12] Marra F, Young F, Richardson K, Marra CA (2013). A meta-analysis of intradermal versus intramuscular influenza vaccines: immunogenicity and adverse events. Influenza Other Respir. Viruses.

[13] Kim YC, Jarrahian C, Zehrung D, Mitragotri S, Prausnitz MR (2012). Delivery systems for intradermal vaccination. Curr. Top. Microbiol. Immunol..

[14] Prausnitz MR (2017). Engineering microneedle patches for vaccination and drug delivery to skin. Annu Rev. Chem. Biomol. Eng..

[15] Nguyen TT, Park JH (2018). Human studies with microneedles for evaluation of their efficacy and safety. Expert Opin. Drug Deliv..

[16] Shin CI, Jeong SD, Rejinold NS, Kim YC (2017). Microneedles for vaccine delivery: challenges and future perspectives. Ther. Deliv..

[17] Marshall S, Sahm LJ, Moore AC (2016). The success of microneedle-mediated vaccine delivery into skin. Hum. Vaccin Immunother..

[18] Forster AH (2020). Safety, tolerability, and immunogenicity of influenza vaccination with a high-density microarray patch: Results from a randomized, controlled phase I clinical trial. PLoS Med..

[19] Taddio A (2012). Survey of the prevalence of immunization non-compliance due to needle fears in children and adults. Vaccine.

[20] Rouphael NG, Mulligan MJ (2017). Microneedle patch for immunization of immunocompromised hosts. Oncotarget.

[21] Skountzou I, Compans RW (2015). Skin immunization with influenza vaccines. Curr. Top. Microbiol. Immunol..

[22] Lee BY (2015). An economic model assessing the value of microneedle patch delivery of the seasonal influenza vaccine. Vaccine.

[23] Badizadegan K, Goodson JL, Rota PA, Thompson KM (2020). The potential role of using vaccine patches to induce immunity: platform and pathways to innovation and commercialization. Expert Rev. Vaccines.

[24] Nakatsukasa A (2017). Potency of whole virus particle and split virion vaccines using dissolving microneedle against challenges of H1N1 and H5N1 influenza viruses in mice. Vaccine.

[25] Quan FS (2013). Long-term protective immunity from an influenza virus-like particle vaccine administered with a microneedle patch. Clin. Vaccin. Immunol..

[26] Quan FS, Kim YC, Compans RW, Prausnitz MR, Kang SM (2010). Dose sparing enabled by skin immunization with influenza virus-like particle vaccine using microneedles. J. Control Release.

[27] Rouphael NG (2017). The safety, immunogenicity, and acceptability of inactivated influenza vaccine delivered by microneedle patch (TIV-MNP 2015): a randomised, partly blinded, placebo-controlled, phase 1 trial. Lancet.

[28] Monto AS (2015). Antibody to influenza virus neuraminidase: an independent correlate of protection. J. Infect. Dis..

[29] Couch RB (2013). Antibody correlates and predictors of immunity to naturally occurring influenza in humans and the importance of antibody to the neuraminidase. J. Infect. Dis..

[30] Monsalvo AC (2011). Severe pandemic 2009 H1N1 influenza disease due to pathogenic immune complexes. Nat. Med..

[31] Bentebibel SE (2013). Induction of ICOS+CXCR3+CXCR5+ TH cells correlates with antibody responses to influenza vaccination. Sci. Transl. Med..

[32] Bentebibel S-E (2016). ICOS+PD-1+CXCR3+ T follicular helper cells contribute to the generation of high-avidity antibodies following influenza vaccination. Sci. Rep..

[33] Koutsakos, M. et al. Circulating T(FH) cells, serological memory, and tissue compartmentalization shape human influenza-specific B cell immunity. *Sci. Transl. Med*. **10**, 10.1126/scitranslmed.aan8405 (2018). 10.1126/scitranslmed.aan8405 29444980

[34] Hirobe S (2015). Clinical study and stability assessment of a novel transcutaneous influenza vaccination using a dissolving microneedle patch. Biomaterials.

[35] Food and Drug Administration. Guidance for industry: clinical data needed to support the licensure of pandemic influenza vaccines. Accessed May 16th, 2021 (2007).

[36] Hobson D, Curry RL, Beare AS, Ward-Gardner A (1972). The role of serum haemagglutination-inhibiting antibody in protection against challenge infection with influenza A2 and B viruses. J. Hyg..

[37] Webster RG, Laver WG (1967). Preparation and properties of antibody directed specifically against the neuraminidase of influenza virus. J. Immunol..

[38] Memoli MJ (2016). Evaluation of antihemagglutinin and antineuraminidase antibodies as correlates of protection in an influenza A/H1N1 virus healthy human challenge model. mBio.

[39] Maier HE (2020). Pre-existing antineuraminidase antibodies are associated with shortened duration of influenza A(H1N1)pdm virus shedding and illness in naturally infected adults. Clin. Infect. Dis..

[40] Wohlbold TJ, Krammer F (2014). In the shadow of hemagglutinin: a growing interest in influenza viral neuraminidase and its role as a vaccine antigen. Viruses.

[41] Gilbert PB (2019). HAI and NAI titer correlates of inactivated and live attenuated influenza vaccine efficacy. BMC Infect. Dis..

[42] Teunissen MB, Haniffa M, Collin MP (2012). Insight into the immunobiology of human skin and functional specialization of skin dendritic cell subsets to innovate intradermal vaccination design. Curr. Top. Microbiol. Immunol..

[43] Athale, S. et al. Influenza vaccines differentially regulate the interferon response in human dendritic cell subsets. *Sci. Transl. Med.* **9**, 10.1126/scitranslmed.aaf9194 (2017). 10.1126/scitranslmed.aaf9194 PMC5484150 28330867

[44] Wilkinson TM (2012). Preexisting influenza-specific CD4+ T cells correlate with disease protection against influenza challenge in humans. Nat. Med.

[45] Sridhar S (2013). Cellular immune correlates of protection against symptomatic pandemic influenza. Nat. Med..

[46] Crotty S (2015). A brief history of T cell help to B cells. Nat. Rev. Immunol..

[47] Sasaki S (2008). Influence of prior influenza vaccination on antibody and B-cell responses. PLoS ONE.

[48] He X-S (2008). Baseline levels of influenza-specific CD4 memory T-cells affect T-cell responses to influenza vaccines. PLoS ONE.

[49] Nougarede N (2014). Nine μg intradermal influenza vaccine and 15 μg intramuscular influenza vaccine induce similar cellular and humoral immune responses in adults. Hum. Vaccin Immunother..

[50] Koutsonanos DG (2015). Enhanced immune responses by skin vaccination with influenza subunit vaccine in young hosts. Vaccine.

[51] Getie-Kebtie M, Sultana I, Eichelberger M, Alterman M (2013). Label-free mass spectrometry-based quantification of hemagglutinin and neuraminidase in influenza virus preparations and vaccines. Influenza Other Respir. Viruses.

[52] Vassilieva EV (2015). Improved immunogenicity of individual influenza vaccine components delivered with a novel dissolving microneedle patch stable at room temperature. Drug Deliv. Transl. Res..

[53] Martínez-Sobrido L (2010). Hemagglutinin-pseudotyped green fluorescent protein-expressing influenza viruses for the detection of influenza virus neutralizing antibodies. J. Virol..

[54] Rajendran, M. et al. Analysis of anti-influenza virus neuraminidase antibodies in children, adults, and the elderly by ELISA and enzyme inhibition: evidence for original antigenic sin. *mBio* **8**, 10.1128/mBio.02281-16 (2017). 10.1128/mBio.02281-16 PMC5362038 28325769

[55] Khurana S (2010). Bacterial HA1 vaccine against pandemic H5N1 influenza virus: evidence of oligomerization, hemagglutination, and cross-protective immunity in ferrets. J. Virol..

[56] Khurana S (2010). Properly folded bacterially expressed H1N1 hemagglutinin globular head and ectodomain vaccines protect ferrets against H1N1 pandemic influenza virus. PLoS ONE.

[57] Khurana S (2011). Recombinant HA1 produced in *E. coli* forms functional oligomers and generates strain-specific SRID potency antibodies for pandemic influenza vaccines. Vaccine.

[58] Verma S (2012). Oligomeric recombinant H5 HA1 vaccine produced in bacteria protects ferrets from homologous and heterologous wild-type H5N1 influenza challenge and controls viral loads better than subunit H5N1 vaccine by eliciting high-affinity antibodies. J. Virol..

[59] Crotty S (2003). Cutting edge: long-term B cell memory in humans after smallpox vaccination. J. Immunol..

