# Supplementary material for: Immunologic mechanisms of seasonal influenza vaccination administered by microneedle patch from a randomized phase I trial
Source: NPJ Vaccines. 2021 Jul 14;6:89. doi: 10.1038/s41541-021-00353-0 (PMC8280206; doi:10.1038/s41541-021-00353-0)
Supplement: Supplementary file 1 — Supplementary Information [file 41541_2021_353_MOESM1_ESM.pdf]

**Supplemental Material**

# Immunologic Mechanisms of Seasonal Influenza Vaccination Administered by Microneedle Patch from a Randomized Phase I Trial

Nadine Rouphael, Lilin Lai, Sonia Tandon, Michele McCullough, Yunchuan Kong, Sarah Kabbani, Muktha Natrajan, Yongxian Xu, Yerun Zhu, Dongli Wang, Jesse O'Shea, Amy Sherman, Tianwei Yu, Sebastien Henry, Devin V McAllister, Daniel Stadlbauer, Surender Khurana, Hana Golding, Florian Krammer, Mark Mulligan, and Mark Prausnitz

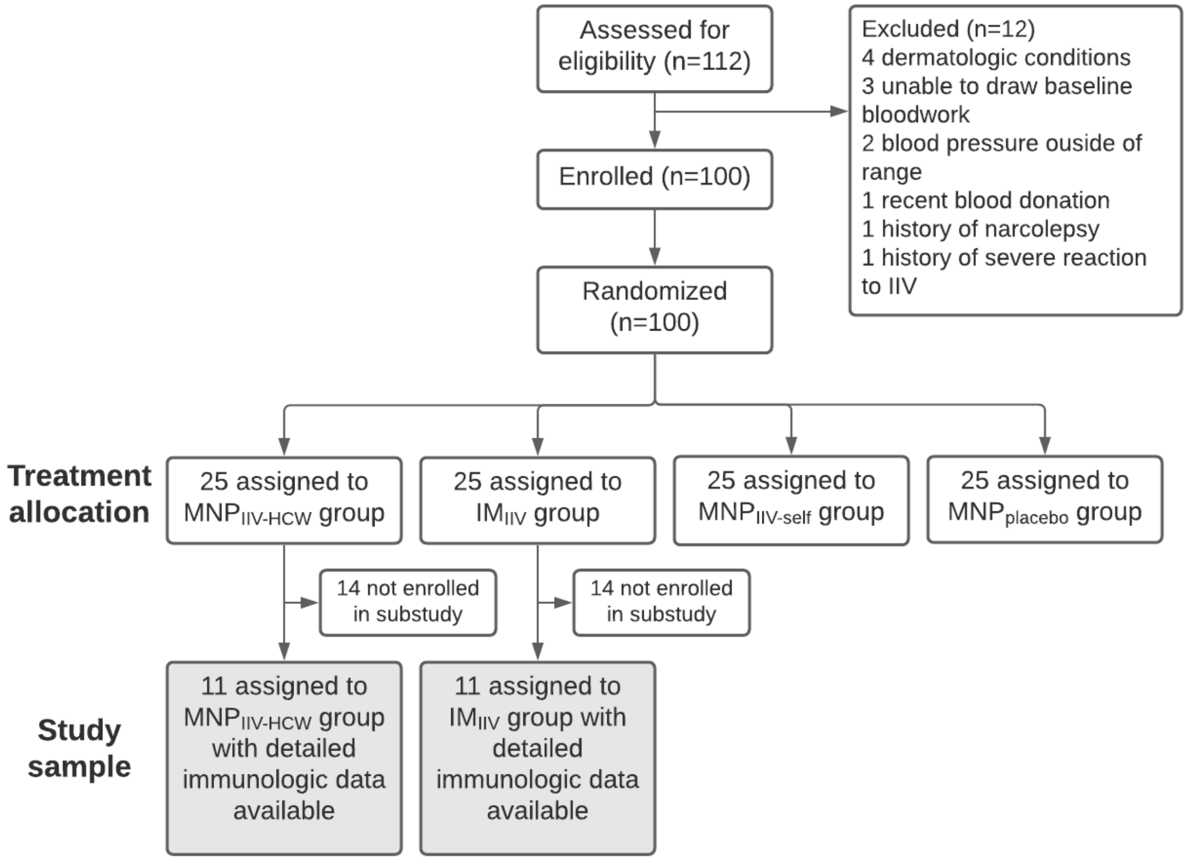


**Supplementary Figure 1.** Selection of study sample for detailed immunologic analysis. $MNP_{IIV\text{-}HCW}$, IIV by MNP; $IM_{IIV}$, IIV by IM injection; $MNP_{IIV\text{-}}$self, IIV by MNP self-administered by study participants; $MNP_{placebo}$, placebo by MNP.

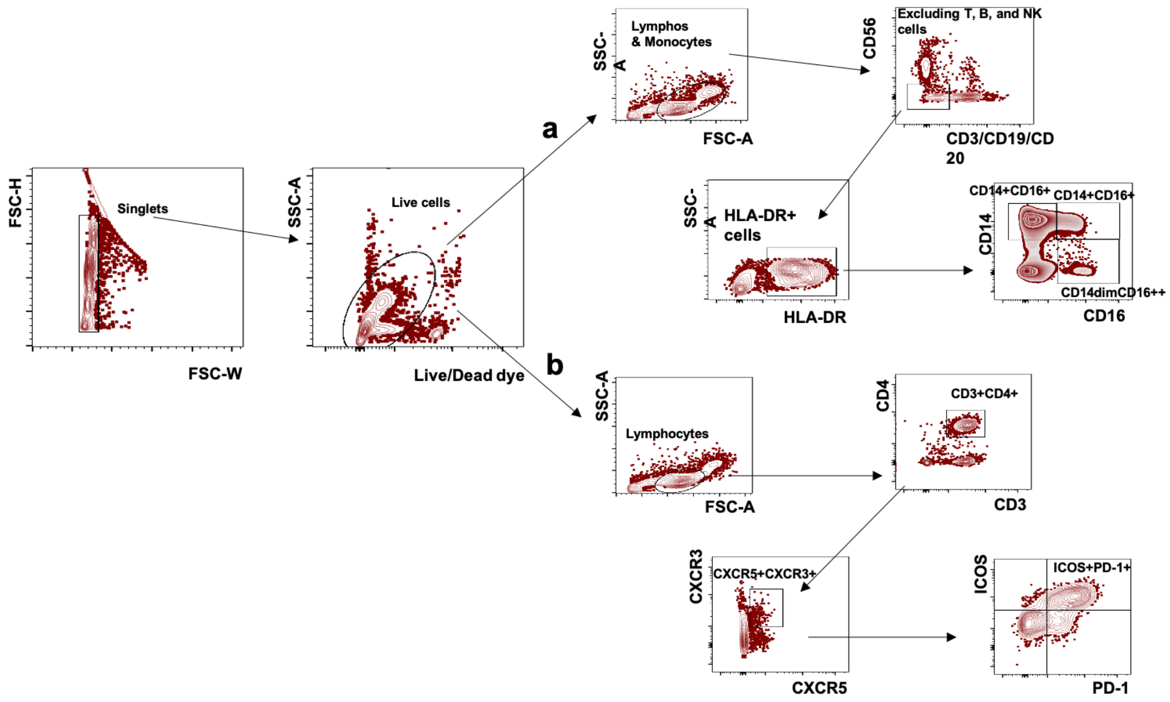


**Supplementary Figure 2.** Gating strategy for monocytes and $cT_{FH}$ cells. Single cells were selected on the basis of forward scatter width and height characteristics (FSC-W and FSC-H). a) Monocytes were identified within the SSC-A-hi FSC-A-hi cells as the CD3-CD19-CD20-CD56-HLA-DR+ population and gated for the CD14+CD16-, CD14+CD16+, and CD14dimCD16++ subsets. b) ICOS+PD1+$cT_{FH}$ cells in PBMCs was identified by ICOS expression on CXCR3+CXCR5+CD4+ T cells.

**Supplementary Table 1.** Hemagglutination inhibition (HAI) geometric mean titers (GMT), seroprotection, and seroconversion at Day 28 and Day 180 following vaccination.

| | A/Christchurch (H1N1) | | | A/Texas (H3N2) | | | B/Massachusetts | | |
|---|---|---|---|---|---|---|---|---|---|
| | **MNP** | **IM** | ***p*** | **MNP** | **IM** | ***p*** | **MNP** | **IM** | ***p*** |
| **GMT Day 0** | 124.4 (63.8, 242.4) | 150.2 (92.4, 244.3) | *0.78* | 54.81 (28.9, 103.7) | 58.4 (28.9, 117.8) | *0.81* | 42.6 (25.1, 72.3) | 51.5 (35.3, 75.0) | *0.53* |
| **GMT Day 28** | 1201.8 (612.9, 2356.4) | 934.1 (530.9, 1643.6) | *0.59* | 248.71 (131.9, 468.9) | 219.3 (129.7, 370.8) | *0.66* | 132.4 (79.2, 221.4) | 80 (59.6, 107.4) | *0.08* |
| **GMT Day 180** | 411.7 (165.0, 1,027.2) | 362.9 (210.7, 625.2) | *0.64* | 90.8 (45.8, 180.0) | 70.5 (36.7, 135.4) | *0.59* | 58.4 (30.9, 110.4) | 80 (59.6, 107.4) | *0.99* |
| **GMT Day 28/Day 0** | 9.7 (3.9, 23.4) | 6.2 (3.2, 12.1) | *0.42* | 4.5 (2.2, 9.3) | 3.8 (1.92, 7.36) | *0.84* | **3.1 (2.3, 4.3)** | **1.6 (1.1, 2.3)** | ***0.009*** |
| **GMT Day 180/Day 0** | 3.3 (1.2, 9.4) | 2.4 (1.14,5.13) | *0.33* | 1.7 (1.2, 2.4) | 1.2 (0.8, 1.7) | *0.29* | 1.37 (1.0, 1.9) | 1.1 (0.9, 1.5) | *0.29* |
| **Seroprotection (Day 28)** | 100% (71.5, 100) | 100% (71.5, 100) | *1.00* | 100% (71.5, 100) | 100% (71.5, 100) | *1.00* | 100% (71.5, 100) | 100% (71.5, 100) | *1.00* |
| **Seroprotection (Day 180)** | 100% (71.5, 100) | 100% (71.5, 100) | *1.00* | 90.9% (58.7, 99.8) | 81.8% (48.2, 97.7) | *0.99* | 81.8% (48.2, 97.7) | 90.9% (58.7, 99.8) | *1.00* |
| **Seroconversion (D28/D0)** | 81.8% (48.2, 97.7) | 81.8% (48.2, 97.7) | *1.00* | 72.7% (39.0, 93.9) | 72.7% (39.0, 93.9) | *1.00* | 54.5% (23.4, 83.3) | 18.2% (2.3, 51.8) | *0.18* |
| **Seroconversion (D180/D0)** | 45.5% (16.7, 76.6) | 27.3% (6.0, 60.9) | *0.66* | 18.2% (2.3, 51.8) | 0% (0.0, 28.5) | *0.48* | 0% (0.0, 28.5) | 0% (0.0, 28.5) | *1.00* |

Values are GMTs with 95% CIs based on normal distribution of log-transformed values. Mann-Whitney Wilcoxon tests were used to compare MNP (n=11) and IM (n=11) groups. For seroprotection and seroconversion, p-values were calculated using chi-square test and 95% CIs are calculated using Clopper-Pearson exact CIs. A two-sided p-value of $< 0.05$ was considered statistically significant.

HAI, Hemagglutination inhibition; GMT, geometric mean titer, GMT; microneedle patch; IM, intramuscular injection; CI, confidence interval.

**Supplementary Table 2.** Neuraminidase inhibition (NAI) geometric mean titers (GMT) at Day 28 and Day 180 following vaccination.

| | H6N1 | | | H6N2 | | | H6NB | | |
|---|---|---|---|---|---|---|---|---|---|
| | **MNP** | **IM** | ***p*** | **MNP** | **IM** | ***p*** | **MNP** | **IM** | ***p*** |
| **GMT Day 0** | **57.1 (32.2, 101.4)** | **111.9 (88.1, 142.1)** | ***0.04*** | 42.4 (24.3, 74.1) | 51.4 (31.6, 83.4) | *0.54* | 137.7 (93.2, 203.5) | 165.5 (83.2, 329.3) | *0.40* |
| **GMT Day 28** | 306.2 (170.3, 550.3) | 169.5 (132.8, 216.2) | *0.15* | 166.7 (100.3, 276.9) | 99.8 (64.9, 153.4) | *0.18* | 230.6 (150.4, 353.6) | 196.3(108.3, 355.6) | *0.90* |
| **GMT Day 180** | 106.6 (58.8, 193.4) | 133.1 (103.8, 170.6) | *0.72* | 84.9 (48.8, 148.1) | 68.5 (40.1, 117.1) | *0.40* | 161.8 (103.4, 253.4) | 157.2 (78.2, 315.8) | *0.99* |
| **GMT Day 28/Day 0** | **5.4 (2.9, 9.7)** | **1.5 (1.2, 1.9)** | ***0.002*** | **3.93 (2.7, 5.8)** | **1.9 (1.6, 2.4)** | ***0.003*** | **1.7 (1.5, 1.9)** | **1.2 (1.1, 1.3)** | ***0.0008*** |
| **GMT Day 180/Day 0** | **1.9 (1.3, 2.7)** | **1.2 (1.0, 1.4)** | ***0.05*** | 2.0 (1.4, 2.9) | 1.3 (1.1, 1.6) | *0.11* | **1.2 (1.0, 1.3)** | **0.9 (0.9, 1.0)** | ***0.02*** |

Values are GMTs with 95% CIs based on normal distribution of log-transformed values. Mann-Whitney Wilcoxon tests were used to compare MNP (n=8) and IM (n=11) groups. A two-sided p-value of < 0.05 was considered statistically significant.

NAI, neuraminidase inhibition; GMT, geometric mean titer; MNP; microneedle patch; IM, intramuscular injection; CI, confidence interval
